# Impacts of Dietary Supplementations of Orange Peel and Tomato Pomace Extracts as Natural Sources for Ascorbic Acid on Growth Performance, Carcass Characteristics, Plasma Biochemicals and Antioxidant Status of Growing Rabbits

**DOI:** 10.3390/ani11061688

**Published:** 2021-06-05

**Authors:** Fawzia A. Hassan, Nabila Elkassas, Ibrahim Salim, Shawky El-Medany, Salama Mostafa Aboelenin, Mustafa Shukry, Ayman E. Taha, Soliman Peris, Mohamed Soliman, Khalid Mahrose

**Affiliations:** 1 Department of By-Products Utilization Research, Animal Production Research Institute, Agricultural Research Center, El-Dokki, Giza 12618, Egypt; fawzia_amer@yahoo.com (F.A.H.); nabilaelkassa77@yahoo.com (N.E.); ibrahim_salim@gmail.com (I.S.); 2 Regional Center for Food and Feed, Agricultural Center, Giza 12618, Egypt; shawky_elmedany@gmail.com; 3 Biology Department, Turabah University College, Taif University, P.O. Box 11099, Taif 21944, Saudi Arabia; s.aboelenin@tu.edu.sa; 4 Department of Physiology, Faculty of Veterinary Medicine, Kafrelsheikh University, KafrEl-Sheikh 33516, Egypt; mostafa_shukry2002@yahoo.com; 5 Animal Husbandry and Animal Wealth Development, Faculty of Veterinary Medicine, Alexandria University, Abis, Alexandria 21944, Egypt; ayman_soma2007@yahoo.com; 6 Animal Production Department, Faculty of Agriculture, Zagazig University, Zagazig 44511, Egypt; soliman_peris@hotmail.com; 7 Clinical Laboratory Sciences Department, Turabah University College, Taif University, P.O. Box 11099, Taif 21944, Saudi Arabia; mmsoliman@tu.edu.sa; 8 Animal and Poultry Production Department, Faculty of Technology and Development, Zagazig University, Zagazig 44511, Egypt

**Keywords:** antioxidants, ascorbic acid, fattening rabbits, orange peel, tomato pomace

## Abstract

**Simple Summary:**

There has been growing interest in using natural feed additives in to enhance animal performance and meat quality for human consumption. Citrus fruit residues can act as potential natural resources of antioxidants, which comprise a considerable quantity of ascorbic acid. Tomato pomace powder has a good nutritional value because of its content of essential amino acids and fatty acids besides its high content of antioxidants. This work examined the impact of dietary orange peel and tomato pomace extract supplementations at level of 200 mg/kg on growth performance, plasma biochemicals, carcass characteristics and antioxidant status of growing male rabbits. Dietary supplementations of orange peel and tomato pomace extracts could effectively improve growth performance, antioxidative status, modulate ascorbic acid level in plasma and meat and lower the plasma total cholesterol.

**Abstract:**

The effect of dietary orange peel (OPE) and tomato pomace extract (TPE) supplementations on growth performance, plasma biochemicals, carcass characteristics and antioxidant status of growing male rabbits were investigated. A total of 96 rabbits (5 weeks old) were distributed into four groups. The first group received untreated pelleted diet (control). The second group was fed a diet containing ascorbic acid (AA; 1.0 g/kg diet), while the third and fourth groups consumed diets supplemented with 200 gm of OPE or (TPE, respectively. Our results indicated that OPE and TPE contained 59, 14.03 mg ascorbic acid/100 g DM, respectively. Growth performance, except feed conversion ratio, and carcass weight were improved by dietary supplementations. Dietary supplementations decreased kidneys, abdominal, back fats and ether extract of meat. Plasma protein and globulin levels were high in rabbits fed AA and TPE-supplemented diets. Low plasma total cholesterol and LDL-cholesterol concentrations were observed in rabbits fed the supplemented diets. Plasma AA was increased in rabbits fed AA and OPE-supplemented diets. Rabbits fed OPE and TPE-supplemented diets had great SOD activity. The best economic efficiency was recorded by rabbits fed the supplemented diets. Dietary supplementations of OPE and TPE could effectively improve growth performance, antioxidative status, modulate AA level in plasma and meat and lower plasma total cholesterol and LDL.

## 1. Introduction

Recently, there has been growing interest in using natural feed additives to enhance animal performance and meat quality for human consumption [1,2,3,4]. Water-soluble vitamins such as vitamin C (L-ascorbic acid or simply ascorbate) are normally synthesized by the rabbit digestive flora and the dietary supplementation of 25–30 mg/rabbit/day may be advisable in cases of the digestive disorders, as after weaning [5]. Furthermore, its activity in neutralizing reactive oxygen species, as a natural free radical scavenger [6,7], through the oxidizing of ascorbic acid to monodehydroascorbate reacts with free radicals [8]. Additionally, ascorbic acid has a beneficial role against toxic effects [9]. 

Citrus fruit residues, these residues are readily available on demand and affordable to the breeders, can act as potential natural resources of antioxidants, which comprise a considerable quantity of ascorbic acid [10]. Orange peel has complexes such as catechol, dimethoxy phenol, cyclohexane, coumarin, acetic acid, stigmasterol, sitosterol and vitamin E which are accountable for its antioxidant feature [11]. Orange peel has high antioxidant activity [12], where the aqueous extract of orange peel powder showed an antioxidant activity of 71.2% [11,12]. Orange peels contain high amount of flavonoids and vitamin C (110.4–127.70 mg/100 g of orange peel on dry basis) [13,14].

Tomato pomace, including peels and seeds, is produced as a by-product in the tomato processing industry [15]. The huge amount generated from commercial tomato processing has caused a severe waste problem [16]. Tomato pomace powder has a good nutritional value because of its content of essential amino acids and fatty acids besides its high content of antioxidants such as flavonoids, phenolic acids, lycopene, carotenoids, vitamins A, C, and E; as well it contains various minerals such as Ca, Cu, Mn, Zn, and Se [17].

Vitamins are organic compounds that are essential for nutrients metabolism, growth, production and animal health [1,18]. Ani and Abel [19] found that AA content of Citrus maxima peel extract was19.34 mg/100 g lower than the value of the present study. In addition, M’hiri et al. [20] reported that orange peel powder had 0.105 ± 0.003 g/100 g dry basis of AA. Sir Elkhatim et al. [13] stated high amount (110.4 mg/100 g) of AA in orange peels. The fine powders of tomato pomace and its peel can be applied as functional feed supplementation [17] through ethanol extraction method. As well tomato by-product had 0.25 g/kg AA [21]. Therefore, the antioxidant activity of tomato peel extract is relatively high [15]. So, OPE and TPE could be alternatives to dietary AA [11,22].

The aim of the current study was to determine how the extract of orange peel and tomato pomace supplementation at level of 200 mg/kg diet influences the rabbit’s growth performance, carcass characteristics, plasma biochemicals, antioxidant status and economic efficiency of growing rabbit diets.

## 2. Materials and Methods

The experimental work of this study was carried out at El-Gemmaza, El-Gharbia Governorate, Experimental Station of Animal Production, Animal Production Research Institute, Agricultural Research Center, Ministry of Agriculture, Egypt (APRI/132429/191214). 

### 2.1. Extract Preparation

Orange peel and tomato pomace were obtained from Kaha Company for preserved foods, EL-Qaliobia Governorate, Egypt. Tomato pomace contains skin and seeds. Orange peel and tomato pomace were extracted with 95% ethanol at a ratio of 1 part of residue: 2 parts of 95% ethanol *w*/*v* for 24 h, the extraction was filtered and the residue was re-extracted two times under the same conditions. The extract was evaporated in a rotary evaporator below 40 °C and freeze-dried in a freeze-dry system until use. 

### 2.2. Experimental Design, Animals and Diets

A total number of 96 of weaned V-line male rabbits at 5 weeks old and have average initial live body weight (603.67 ± 11.77 g) were randomly assigned to four experimental groups (24/each in 3 replicates) in a complete simple randomized design. The first experimental group received daily untreated pelleted diet (control). The second group received diet containing ascorbic acid (AA) at 1.0 g/kg diet. The third and fourth groups received daily pelleted diet supplemented with either 200 gm of ORE or 200 gm of TPE, respectively. The experimental period continued for 8 weeks. Rabbits were individually housed in galvanized wire cages (Dimensions of 60 × 40 × 35 cm) until marketing at 14 weeks of age. All rabbits were fed pelletized feed ad libitum, fresh water was automatically available all the time by stainless steel nipples fixed in each cage. Feed ingredients and chemical composition of experimental diets (%DM basis) are shown in Table 1. The experimental diets were formulated to meet the recommended nutrient requirements of growing rabbits according to [23]. The ascorbic acid content of OPE and TPE is presented in Table 2.

All rabbits were kept under the same management, hygienic and environmental conditions. Live body weight (BW) was determined weekly throughout the experimental period, and body weight gain (BWG) was calculated. Feed consumption (FC) was determined precisely and calculated as grams/rabbit/day (during the all experimental period). Unused feed from each cage was collected daily, weighed and taken into consideration for the calculation of FC, accordingly, feed conversion ratio was computed (FCR; g feed/g gain).

### 2.3. Slaughtering and Carcass Characteristics

At the end of the experimental period, six male rabbits/each group were randomly taken, fasted for 12 h, individually weighed and immediately slaughtered. Slaughter procedure and carcass analysis were carried out as early described [24,25]. After complete bleeding, pelt, viscera’s and tail were removed then the carcass and its components were weighed as edible parts. Heart, liver, kidneys, spleen, cecum, kidneys fat, abdominal fat and back fat were also weighed as percentage of pre-slaughter weight. Dressing percentage was calculated by dividing the hot dressed carcass weight by pre-slaughter weight and expressed as a percentage.

### 2.4. Chemical Analysis

Experimental diet and meat samples of rabbits were analyzed for dry matter (DM), organic matter (OM), crude protein (CP), ether extract (EE), crude fiber (CF), and nitrogen free extract (NFE) and ash according to the methods of AOAC [26]. Ascorbic acid was assayed using HPLC, according to Danish official method [27].

### 2.5. Blood Samples and Determination of Plasma Biochemicals

Blood samples were collected during slaughtering to determine blood biochemicals and centrifuged at 3000 r.p.m. for 15 min to separate blood plasma. Plasma total protein, albumin, total cholesterol, LDL and HDL-cholesterol, vLDL, triglycerides, total lipids, aspartate aminotransferase (AST), alanine aminotransferase (ALT) and ascorbic acid were colorimetrically determined using profitable kits (purchased from Bio-diagnostic, Egypt) as stated by the manufacturers’ guidelines. Plasma globulin concentration was considered by the difference between total protein and albumin, and then albumin/globulin ratio (A/G ratio) was calculated. Blood antioxidant constituents as superoxide dismutase (SOD) and total antioxidant capacity (T-AOC) were inspected by colorimetric procedure consuming saleable kits (Bio-diagnostic, Cairo, Egypt).

### 2.6. Economic Efficiency

To determine the economic efficiency of the experimental diets for BWG, the costs of the feed required for producing one kilogram of BWG was estimated. The cost of the experimental diets was calculated according to the price of different ingredients prevailing at local market as well as the price of tested materials at the time of experimentation (2021). Economic efficiency was calculated as a ratio between the return of BWG and the cost of FC. 

### 2.7. Statistical Analysis

The obtained data were statistically analyzed using the general linear model procedure of SAS^®^ Software Statistical Analysis (Cary, NC, USA) [28]. Variances among averages were verified [29]. All results were analyzed using this model: Yij = μ + Ti + Eij; where: Yij = the observation of ij; μ = the overall mean; Ti = the effects of i (treatments) and Eij = the experimental random error.

## 3. Results

The obtained results found in Table 2 indicated that OPE contained 59 mg/100 g DM of vitamin C, while TPE had 14.03 mg/100 g DM of ascorbic acid.

The results presented in Table 3 illustrated that dietary supplementation of AA, OPE and TPE significantly (*p* < 0.05) improved final BW, BWG during 5–13 weeks of age and FC through 5–9, 9–13 and 5–13 weeks of age compared to the control group. However, FCR did not affect by the different dietary supplementations at all experimental periods. 

Carcass characteristics of rabbits as affected by dietary OPE and TPE are presented in Table 4. The gained results revealed that rabbits fed diets supplemented with AA, OPE and TPE showed greater hot carcass weight (*p* < 0.05) than the control group. It is clear to notice that rabbits fed OPE and TPE-supplemented diets exhibited significant (*p* < 0.001) reductions in kidney fat and abdominal fat when compared with their counterparts. Rabbits of the control group presented the greatest (*p* < 0.01) back fat when compared with the other groups. There were insignificant differences among the tested groups in the other carcass traits studied. 

Data concerning the effects of AA, OPE and TPE supplementations on the chemical composition of rabbit meat are shown in Table 5. The results revealed that dietary supplementations of AA, OPE and TPE significantly (*p* < 0.001) decreased EE content compared to the control group. Regarding ascorbic acid concentration, dietary supplementation of AA, OPE and TPE significantly (*p* < 0.01) increased ascorbic acid concentration in hind leg meat of rabbits compared to the control group. Rabbits fed diets supplemented with AA had the highest concentration (0.34 mg/100 g DM) of ascorbic acid, while the control group was the lowest one (0.006 mg/100 g DM). On the other hand, there were insignificant differences in DM, CP and Ash contents among all the experimental groups. 

As shown in Table 6, plasma total protein and globulin levels were higher (*p* < 0.05) in rabbits fed diet supplemented with AA and TPE compared to the control and OPE groups. In addition, lower (*p* < 0.001) plasma total cholesterol and LDL-cholesterol concentrations were observed in rabbits fed diet containing AA, OPE and TPE diets compared to rabbits given the control diet, while non-significant differences in albumin, triglycerides, vLDL and total lipids levels were observed among all tested groups. Data concerning ascorbic acid content indicated that a significant (*p* < 0.05) increase in plasma ascorbic acid level in rabbits fed diets supplemented with AA and OPE by 25.71 and 26.91% compared to the control and TPE groups. Meantime, an insignificant difference was noticed in ascorbic acid concentration between rabbits fed diet included TPE and the other experimental groups.

Regarding the antioxidative status, rabbits fed diets supplemented with OPE and TPE had greater (*p* < 0.05) SOD activity than the control, while rabbits of AA groups had intermediate activity without significant differences. There were insignificant differences among the tested groups in T-AOC level.

The effects of dietary supplementation of AA, OPE and TPE on economic efficiency are shown in Table 7. The greatest economic efficiency and relative economic efficiency were exhibited by rabbits fed diet supplemented with AA followed by OPE and TPE. Dietary supplementation with AA, OPE and TPE of rabbits proved to be more economic than the control group. Rabbits fed 200 mg OPE achieved the highest net revenue followed by those fed 200 mg TPE and 1 gm AA.

## 4. Discussion

The main focus of the present investigation was to verify the ability of dietary supplementations of OPE and TPE to boost growth performance, carcass characteristics, plasma biochemicals, antioxidant status and economic efficiency of growing rabbit.

The current findings regarding growth performance are consistent with those of Abdel-latif et al. [30] who reported that dietary supplementation of AA at level of 200 mg in rabbits led to an improvement in BWG and FCR. In addition, Hamza [31] demonstrated that dietary AA supplementation in rabbits at levels of 0.5, 1.0 and 1.5 g/kg diet increased final BW, BWG and FCR, while FI was not changed. In this direction, Elwan et al. [32] stated that diets supplemented with 1–2% tomato powder enhanced BW, BWG and feed efficiency and reduced FC in fattening rabbits. Peiretti et al. [33] stated that rabbits fed diets included tomato pomace at level of 3% enhanced final BW compared to the control group. On the contrary, Elkomy et al. [34] reported that there was no impact on BW and FI was decreased when rabbits received diets containing dried tomato pomace at levels of 10, 15 and 20%. It was clearly shown that OPE and TPE contained an appreciable amount of AA which enhanced growth performance of rabbits and they could be considered as natural growth promoters. Ascorbic acid promoted growth and counteracted infections by pathogenic bacteria and viruses [35]. In addition, AA is an antioxidant against free radicals and prevents cell damage [7]. Additionally, TPE may be used as antioxidant supplements due to its content of AA and the antioxidant minerals especially Zn and Se [15] which are important for antioxidant enzymes as well it is a rich source of phenols which had scavenging ability on free radicals due to their hydroxyl groups in tomato peels [36]. The increase in FC showed by rabbits consumed diets supplemented with OPE and TPE might have occurred owing to their ability to enhance palatability and flavor of the diets which consequently improved FI and growth performance accordingly. As regards plant extracts rich in polyphenols, it has been shown that digestive secretions, such as saliva and digestive enzymes, increase the absorption and utilization of nutrients which, in turn, increase the growth of animals [37]. Tomato by-products are rich sources of amino acids especially lysine, pigments such as β-carotene and lycopene, and ascorbic acid, vitamin E and micronutrients [32,38]. El-Desoukey et al. [39] indicated that OPE has powerful antibacterial effect against enteric pathogens which can lead to an enhancement of the digestive and immune systems.

Carcass characteristics results herein are in accordance with those found by Elazab et al. [40] and Elkomy et al. [34] who demonstrated that carcass characteristics did not differ when rabbits fed diets containing 10–20% tomato pomace. Furthermore, Hosseini-Vashan et al. [41] stated that broilers fed diet supplemented with dried tomato pomace at levels of 3–5% had lower relative weight of abdominal fat than the control group. Hamza [31] noticed that dietary AA supplementation in rabbits at levels of 0.5–1.5 g/kg diet showed did not change dressing percentage and relative weights of liver, kidney, heart, lungs and spleen organs. Conversely, Alefzadeh et al. [42] found that dietary orange peel powder at 2% reduced weight of most of the carcass parts in broilers. Sherif [43] concluded that AA supplementation at a level of 0.5 g/kg diet was positively affected carcass yield and total edible parts percentages of rabbits. Based on the present results, The use of OPE and TPE supplementations in the rabbit diets had beneficial effects in reducing abdominal, kidneys and back fat in rabbits carcass and that may be due to the that ascorbic acid has an antioxidant feature may prevent lipid peroxidation [44]. Additionally, AA participates in the hydroxylation of cholesterol to bile acids and reduced its level in the blood stream and tissues. As earlier studies have shown, AA plays a critical role in the carnitine biosynthesis, an essential cofactor in fatty acid oxidation [45]. Citrus peel extract was also reported to regulate lipoprotein metabolism in rats by increasing β-oxidation and lipolysis in the adipose tissue of rats [46]. Tomato by-products are rich in multiple compounds with have antioxidant properties such as carotenes, lycopene, phenolic compounds, flavonoids, ascorbic acid and vitamin A [38,47].

Concentrations of AA were low in the current work since vitamin C is soluble in water and it is not stored in the body but rapidly excreted [1]. Similar findings were also described by Elazab et al. [40] who indicated that rabbits fed diets containing 10–20% of tomato pomace did not differ significantly in meat content of crude protein, fat and ash compared to the control group. Dietary inclusion of tomato pomace at 0.5% did not affect the proportion of most of the fatty acids in the tissues of pig [48]. Our results were confirmed by Horváth and Babinszky [49] who mentioned that ascorbic acid is actively transported into tissues, but during stress, ascorbic acid is produced and consumed rapidly and its amount synthesized fall below animal requirements [50]. Dietary supplementation of vitamin C may cause an increase in the vitamin C of rabbit’s meat and reducing the oxidation of the lipids [51]. On the contrary, Mourão et al. [52] reported that broiler chickens received diets supplemented with 50 and 100 g/kg citrus pulp had no impact on total lipid content of meat.

Our outcomes regarding blood biochemicals are in harmony with those obtained by Sayed and Abdel-Azeem [53] who showed that serum protein, albumin and globulin of rabbits were not affected by different levels of dried tomato seeds (10–30%). Sherif [43] concluded that rabbits fed diet containing 0.5 g vitamin C had low plasma cholesterol levels. The low levels of plasma cholesterol and LDL-cholesterol in our work may be associated with the antioxidant ability of ascorbic acid which is mainly attributed to its role as a cofactor for the enzyme 7-alpha-hydroxylase, the rate limiting enzyme in the transformation of cholesterol to bile acids [54]. It has been verified that ascorbic acid is able to intercept reactive oxygen species in the aqueous phase of plasma, thereby reducing plasma lipid peroxide levels and thus inhibiting oxidation of LDL and removing from blood [55]. Citrus peel extract regulates lipid and triglyceride accumulation [56]. Moreover, Ascorbic acid is a remarkable antioxidant and can decrease the hypercholesterolemia development in rabbits [8]. In this regard, Abdulameer [57] observed that the serum cholesterol and triglycerides concentrations were not altered by dietary vitamin C supplementation and sweet orange peel at levels of 500ppm and 1–2% in broilers. 

The existing findings referred to that rabbits ascorbate status can be modulated by ascorbic acid intake, which is absorbed in the gastrointestinal tract then circulates freely in plasma [58]. Similar findings were observed by Khaled et al. [59] who found that adult male rabbits fed diet containing 40 mg/kg/BW/day for 12 weeks can improve the antioxidant status by reducing free radicals. Results of the present investigation were previously confirmed by those reported with Andres et al. [47] who demonstrated that tomato by-products are rich in multiple compounds with antioxidant properties such as carotenes, lycopene, phenolic compounds, flavonoids, ascorbic acid and vitamin A [17,47]. In addition, orange peel extract exhibited variable antioxidant activity [11]. Additionally, according to the determination of AA in this study, OPE and TPE contain 59 and 17.34 mg/100 g DM, respectively. Recent works have shown that extracts of orange peel show high activity in antioxidants [60]. It is noteworthy that the OPE and TPE had protective antioxidant activities and maintain the body functions. SOD is an enzymatic antioxidant that catalyzes the conversion of O^2−^ to H_2_O_2_ and helps maintain the redox balance by diffusing the superoxide [61].

It could be concluded that there was an improvement of the economic efficiency of diets containing AA, OPE and TPE by 7.81, 6.21 and 4.54, respectively, compared with the control one, due to the improvement of the performance of rabbits. The addition of OPE and TPE as alternative sources of AA enhance the antioxidant status of rabbits [11,17] and lead optimizing the dietary intake of antioxidants such as ascorbic acid.

## 5. Conclusions

In conclusion, dietary supplementation of orange peel and tomato pomace seemed to be much more effective as alternative sources of ascorbic acid. Moreover, they could effectively improve growth performance, antioxidative status, modulate the AA level in plasma and meat and lower the plasma cholesterol.

## Figures and Tables

**Table 1:** Feed ingredients and chemical composition of experimental diets (% DM basis).

Feed Ingredients (%)	Control	Experimental Diets (%)	
		Chemical Composition (%DM Basis)	
Soybean meal (44%CP)	20.9	DM	87.88
Barley	32.0	OM	90.84
Wheat bran	9.20	CP	17.02
Clover hay	31.0	CF	13.28
Molasses	3.00	EE	1.98
Limestone	0.70	NFE	58.56
Di- Ca- phosphate	2.20	Ash	9.16
DL-Methionine	0.40	Methionine ^2^	0.68
NaCl	0.30	Methionine + cysteine ^3^	0.76
Vit.-Min. premix ^1^	0.30	Lysine ^4^	0.99
Total	100	Calcium ^5^	1.27
		Available Phosphours ^6^	0.54
		Digestible energy (Kcal/Kg DM; calculated) ^7^	2867.7

^1^ Mineral and vitamin mixture supplied per kg of diet: Vitamin A 10,000 IU, Vitamin D3, 1800 UI; Vitamin E, 15 mg; vitamin K3, 4.5 mg; Vitamin B1, 0.5 mg; Vitamin B2, 4 mg; Vitamin B12, 0.001 mg; Folic acid, 0.1 mg; Pantothenic acid, 7 mg; Nicotinic acid, 20 mg; I, 1 mg; Mn, 60 mg; Cu, 5.5 mg, Zn, 75 mg; Fe, 40 mg; Co, 0.3 mg; Se, 0.08 mg; Robenidine, 52.8 mg. ^2–7^ Calculated on the basis of the ingredients composition.

**Table 2:** Ascorbic acid content of orange peel and tomato pomace extracts.

Vitamin	Orange Peel Extract	Tomato Pomace Extract
Ascorbic acid (mg/100 g DM)	59	14.03

**Table 3:** Growth performance of growing rabbits fed diets supplemented with AA, OPE and TPE during different ages.

Items	Experimental Diets				*p*-Value
	Control	AA	OPE	TPE	
Initial body weight (g/rabbit)	604.33 ± 27.39	602.00 ± 24.74	604.66 ± 25.39	604.67 ± 18.12	0.9998
Final body weight (g/rabbit)	1872.33 ^b^ ± 44.75	2040.69 ^a^ ± 49.39	2087.00 ^a^ ± 30.86	2089.33 ^a^ ± 27.40	0.0005
**Body weight gain (**g/rabbit**)**					
Weeks 5–9	20.84 ^b^ ± 1.62	22.71 ^ab^ ± 1.09	24.74 ^a^ 0.71	24.26 ^a^ ± 0.73	0.066
Weeks 9–13	19.75 ± 1.56	22.93 ± 1.16	22.56 ± 1.08	23.01 ± 0.77	0.170
Weeks 5–13	20.13 ^b^ ± 0.82	22.84 ^a^ ± 0.77	23.53 ^a^ ± 0.59	23.57 ^a^ ± 0.36	0.0019
**Feed consumption (**g/rabbit/day**)**					
Weeks 5–9	70.03 ^c^ ± 0.58	75.30 ^b^ ± 1.34	82.83 ^a^ ± 0.79	84.50 ^a^ ± 1.15	<0.0001
Weeks 9–13	92.13 ^b^ ± 0.99	94.88 ^b^ ± 1.26	98.60 ^a^ ± 1.35	99.24 ^a^ ± 1.39	0.0005
Weeks 5–13	82.31 ^c^ ± 0.62	86.18 ^b^ ± 1.17	91.59 ^a^ ± 0.95	92.69 ^a^ ± 0.98	<0.0001
**Feed conversion ratio (**g feed/g gain**)**					
Weeks 5–9	3.36 ± 0.39	3.32 ± 0.17	3.35 ± 0.09	3.48 ± 0.17	0.7000
Weeks 9–13	4.66 ± 0.47	4.14 ± 0.28	4.37 ± 0.24	4.31 ± 0.15	0.2300
Weeks 5–13	4.09 ± 0.17	3.77 ± 0.19	3.89 ± 0.10	3.93 ± 0.07	0.4859

AA = ascorbic acid, OPE = orange peel Extract, TPE = tomato pomace extract. ^a,b^ Means with different superscripts in each raw differ significantly (*p* < 0.05).

**Table 4:** Effect of dietary supplementation of AA, OPE and TPE on carcass characteristics of growing rabbits at 13 weeks of age.

Items	Experimental Diets				*p*-Value
	Control	AA	OPE	TPE	
Pre-slaughter weight (g)	1913.3 ± 43.72	2066.7 ± 79.63	2140.0 ± 73.65	2158.3 ± 119.03	0.2280
Hot carcass weight (g)	1136.67 ^b^ ± 33.33	1305.0 ^a^ ± 40.10	1363.33 ^a^ ± 31.79	1278.33 ^a^ ± 31.66	0.0015
Dressing (%)	59.41 ± 1.28	63.14 ± 1.77	63.71 ± 0.86	59.23 ± 3.10	0.3104
Liver (%)	3.93 ± 0.59	5.26 ± 0.58	3.81 ± 0.26	3.88 ± 0.16	0.1410
Heart (%)	0.35 ± 0.07	0.39 ± 0.10	0.31 ± 0.07	0.31 ± 0.08	0.8994
Kidneys (%)	0.87 ± 0.10	0.82 ± 0.11	0.77 ± 0.07	0.70 ± 0.03	0.5670
Spleen (%)	1.62 ± 0.59	1.98 ± 0.30	1.94 ± 0.21	1.81 ± 0.43	0.9226
Cecum (%)	16.48 ± 0.90	13.34 ± 0.41	13.36 ± 1.83	11.99 ± 0.27	0.0795
Kidneys fat (%)	0.93 ^a^ ± 0.11	0.66 ^b^ ± 0.08	0.47 ^c^ ± 0.03	0.44 ^c^ ± 0.07	0.0006
Abdominal fat (%)	0.92 ^a^ ± 0.08	0.62 ^b^ ± 0.02	0.11 ^c^ ± 0.02	0.17 ^c^ ± 0.04	0.0003
Back fat (%)	0.54 ^a^ ± 0.04	0.46 ^b^ ± 0.003	0.38 ^bc^ ± 0.01	0.33 ^c^ ± 0.04	0.0054
Edible giblets (%)	5.16 ± 0.54	6.47 ± 0.58	4.89 ± 0.40	4.88 ± 0.25	0.1223
Total edible parts (%)	64.62 ± 1.68	69.69 ± 1.78	68.65 ± 0.99	64.48 ± 3.32	0.259
Total non-edible parts (%)	35.38 ± 1.69	30.31 ± 1.79	31.35 ± 0.99	35.52 ± 3.32	0.2594

AA = ascorbic acid, OPE = orange peel extract, TPE = tomato pomace extract. Edible giblets (%) = (liver (g) + kidney (g) + heart (g)/pre-slaughter weight (g)]*100%; total edible parts (%) = (carcass weight (g) + weight of edible giblets (g)/pre-slaughter weight (g)*100%. ^a,b,c^ Means with different superscripts in each raw differ significantly (*p* < 0.05).

**Table 5:** Effect of dietary AA, OPE and TPE supplementation on the chemical composition of rabbits meat.

Items		Experimental Diets			
	Control	AA	OPE	TPE	*p*-Value
DM	26.92 ± 0.23	26.65 ± 0.15	26.89 ± 0.10	26.50 ± 0.05	0.2425
CP	22.20 ± 0.11	22.17 ± 0.17	22.51 ± 0.24	22.74 ± 0.08	0.1585
EE	3.43 ^a^ ± 0.03	2.92 ^b^ ± 0.07	2.62 ^bc^ ± 0.21	2.33 ^c^ ± 0.03	0.0004
Ash	1.29 ± 0.12	1.57 ± 0.06	1.76 ± 0.33	1.43 ± 0.04	0.3447
Ascorbic acid (mg/100 g DM)	0.006 ^d^ ± 0.003	0.379 ^a^ ± 0.03	0.340 ^b^ ± 0.11	0.313 ^c^ ± 0.05	0.0018

AA = ascorbic acid, OPE = orange peel extract, TPE = tomato pomace extract. ^a,b,c,d^ Means values with the same letter within the same row did not differ significantly (*p* > 0.05).

**Table 6:** Effect of dietary supplementation of AA, OPE and TPE on plasma parameters and antioxidative status of the growing rabbits.

Items	Supplementations (mg/kg DM)				
	Control	AA	OPE	TPE	*p*-Value
**Plasma parameters**					
Total protein (g/dL)	5.53 ^b^ ± 0.025	6.40 ^a^ ± 0.003	5.61 ^b^ ± 0.08	6.25 ^a^ ± 0.006	0.0001
Albumin (g/dL)	3.84 ± 0.15	3.66 ± 0.01	3.58 ± 0.52	3.23 ± 0.003	0.489
Globulin (g/dL)	1.69 ^c^ ± 0.13	2.74 ^ab^ ± 0.01	2.03 ^bc^ ± 0.46	3.02 ^a^ ± 0.01	0.0151
Total cholesterol (mg/dL)	121.69 ^a^ ± 1.28	11.20 ^b^ ± 0.41	103.37 ^c^ ± 2.27	102.22 ^c^ ± 1.48	<0.0001
Triglycerids (mg/dL)	123.0 ± 0.62	96.62 ± 0.31	97.15 ± 1.30	75.88 ± 7.86	0.3666
LDL (mg/dL)	89.29 ^a^ ± 1.24	80.11 ^b^ ± 0.04	68.37 ^bc^ ± 8.08	63.32 ^c^ ± 0.32	0.0081
VLDL (mg/dL)	22.93 ± 1.76	21.00 ± 1.70	19.43 ± 4.26	15.18 ± 5.56	0.5332
Total lipids (mg/dL)	156.01 ± 25.05	144.24 ± 16.43	127.10 ± 10.74	115.19 ± 6.58	0.3590
Ascorbic acid (mg/L)	157.26 ^b^ ± 0.63	197.69 ^a^ ± 0.58	199.61 ^a^ ± 0.88	174.55 ^ab^ ± 0.29	0.060
**Antioxidative status**					
T-AOC (mmol/L)	1.28 ± 0.006	1.33 ± 0.003	1.31 ± 0.007	1.35 ± 0.02	0.076
SOD (u/L)	28.20 ^b^ ± 0.22	30.30 ^ab^ ± 0.07	33.87 ^a^ ± 1.03	34.19 ^a^ ± 2.04	0.017

^a,b,c^ Mean values with the same letter within the same row did not differ significantly (*p* > 0.05). T-AOC = total antioxidant capacity (mmol/L), SOD = Superoxide dismutase (U/L).

**Table 7:** Effect of dietary treatments on economic efficiency of rabbits diets.

Items	Experimental Diets			
	Control	AA	OPE	TPE
Initial weight (Kg)	0.60	0.60	0.60	0.60
Final weight (Kg)	1.87	2.04	2.09	2.09
Average total weight gain/rabbit (kg)	1.27	1.44	1.48	1.49
Total revenue/rabbit (USD) ^1^	4.04	4.59	4.73	4.74
Total feed intake/rabbit (kg) ^2^	4.61	4.83	5.13	5.19
Price of feeding/kg (USD)	0.31	0.32	0.31	0.31
Total feed cost/rabbit (USD)	1.43	1.54	1.60	1.62
Net revenue/rabbit (USD)	2.62	3.05	3.13	3.11
Economic efficiency(EE) ^3^	1.84	1.98	1.95	1.92
Relative economic efficiency (REE) ^4^	100.00	107.81	106.22	104.54

^1^ Price of one Kg/live body weight on selling was USD 3.19. ^2^ Net revenue = Price of rabbit (USD)—Total feed cost (USD). ^3^ Economic efficiency = Net revenue/Total feed cost (USD). ^4^ Relative economic efficiency (REE) = EE of treatments/EE of the control (assuming that the relative economic efficiency of the control diet is 100).

## Data Availability

The data that support the findings of this study are available on request from the corresponding author. The data are not publicly available due to privacy or ethical restrictions.
